# Electromyography of the muscle spindle

**DOI:** 10.1038/s41598-022-08239-4

**Published:** 2022-03-10

**Authors:** Juhani V. Partanen, Jukka Vanhanen, Sara K. Liljander

**Affiliations:** 1 Department of Clinical Neurophysiology, University of Helsinki and Helsinki University Hospital, P.O. Box 800, 00029 Hus, Finland; 2 Department of Neuroscience and Biomedical Engineering, Aalto University School of Science, P.O. Box 12200, 00076 Aalto, Finland

**Keywords:** Neuroscience, Neuronal physiology, Medical research

## Abstract

In needle electromyography, there are two spontaneous waveforms, miniature end plate potentials and “end plate spikes”, appearing usually together. Miniature end plate potentials are local, non-propagating postsynaptic waves, caused by spontaneous exocytosis of acetylcholine in the neuromuscular junction. The prevailing hypothesis states that “end plate spikes” are propagated postsynaptic action potentials of muscle fibers, caused by presynaptic irritation of the motor nerve or nerve terminal. Using several small concentric needle electrodes in parallel with the muscle fibers, most “end plate spikes” are strictly local or propagating for 2–4 mm. At the end plate zone, there are miniature end plate potentials without “end plate spikes”. Local “end plate spikes” are junctional potentials of intrafusal gamma neuromuscular junctions of the nuclear bag fibers, and propagated “end plate spikes” are potentials of nuclear chain muscle fibers of muscle spindles. Miniature end plate potentials without “end plate spikes” at the end plate zone derive from alpha neuromuscular junctions. These findings contrast with the prevailing hypothesis. The history of observations and different hypotheses of the origin of end plate spikes are described.

## Introduction

In 1941 very short spontaneous potentials were described in electromyography (EMG) of the human extensor communis digitorum muscle^1^. These potentials were also observed in clinical EMG, and it was conjectured that these potentials were derived from nerve filaments since they were usually associated with particularly acute pain^2^. Thus, the term “nerve potentials” was suggested. The same potentials were also described as “protracted irregular activity”. The nature of firing, activations, and waveforms was very accurately described^3^. This “distinctive type of electric activity” was also studied experimentally and found that it has two components, first a low-amplitude component characterized as “the sound of a seashell”, and a second component, which consists of a variable number of spike potentials, usually with an irregular rhythm^4^. This activity was observed in small active sites, which could be marked with iron dots. This activity could also be observed in many animals, including the rat, guinea pig, cat, dog, rabbit, goat, and monkey^4,5^.

Most often the two were combined as sharp spikes on an irregular baseline^4,5^. In man, this activity was observed in 5 to 10% of routine needle insertions in a normal muscle^4,5^. Another hypothesis stated that these spike potentials, “spontaneous diphasic potentials”, originated in the muscle fibers when several synchronous miniature end plate potentials attained an amplitude sufficient to elicit a propagated response^6^. Finally, it was shown that “nerve potentials” were postsynaptic^7^. They were considered to be caused by needle irritation of the motor nerve and recorded postsynaptically. Thereafter, these potentials were called “end plate spikes”^7,8^. But there exists also a hypothesis about the intrafusal origin of end plate spikes^9,10^. Miniature end plate potentials may be observed in both extrafusal^5^ and intrafusal^11^ neuromuscular junctions of muscle fibers. Motor junctional or action potentials are observed in muscle spindles^11^. These potentials may be seen as local and propagated end plate spikes in needle EMG^10^. The rationale of this study was to present convincing evidence that “end plate spikes” are actually fusimotor potentials, consisting of the activity of intrafusal nuclear bag and nuclear chain muscle fibers. The different hypotheses of the origin of end plate spikes are described.

## Material and methods

**Figure 1:**
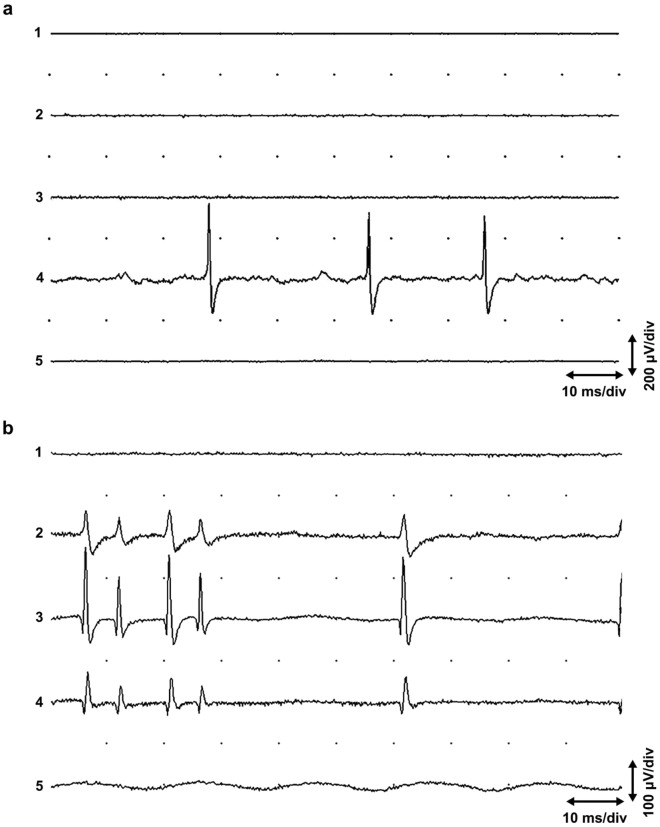
(**a**) Local end plate spikes. Five-channel needle EMG of the extensor carpi radialis muscle showing non-propagating “end plate spikes” with end plate noise in channel 4. The five parallel EMG electrodes (channels 1 − 5) were placed about 2 mm apart in a single fasciculus of the muscle. (**b**) End plate spikes propagating for a short distance (from^39^, with permission). The electrodes as in (**a**).

**Figure 2:**
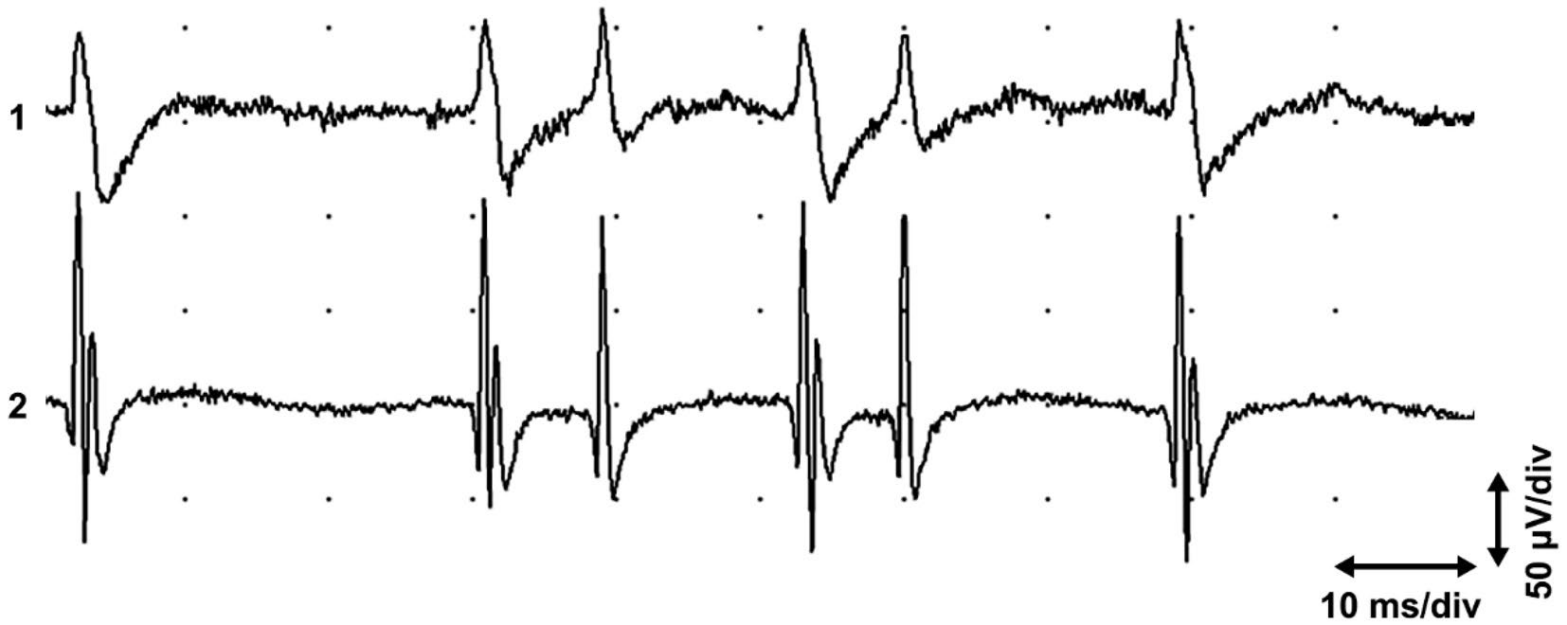
End plate spikes with one or two peaks in the extensor carpi radialis longus muscle. Two concentric parallel EMG needle electrodes in a fasciculus, interelectrode distance 2 mm. Two sequences of propagating “end plate spikes”, one of which shows one-peaked potential in channel 1 and two-peaked potential in channel 2, but the other shows one-peaked potential in both channels.

**Figure 3:**
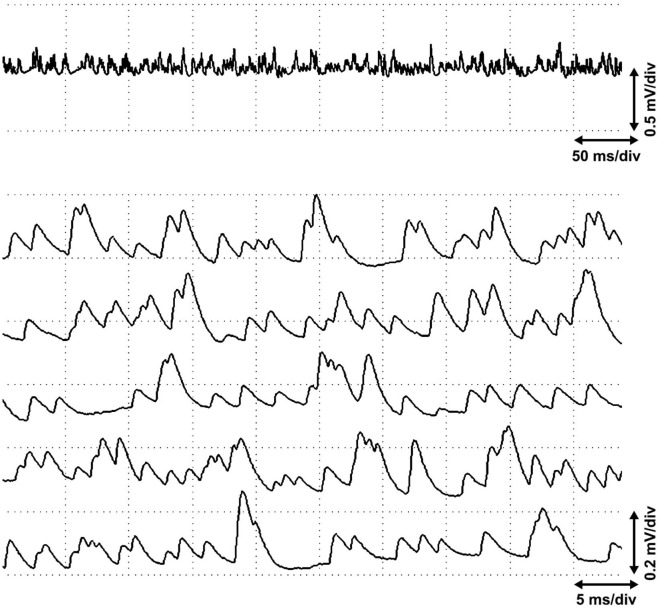
A long sequence of miniature end plate potentials without end plate spikes. Numerous large and partially stratified miniature end plate potentials at the end plate zone of the biceps brachii muscle without any elicited postsynaptic action potentials (cf.^6^) in an active site without end plate spikes.

**Figure 4:**
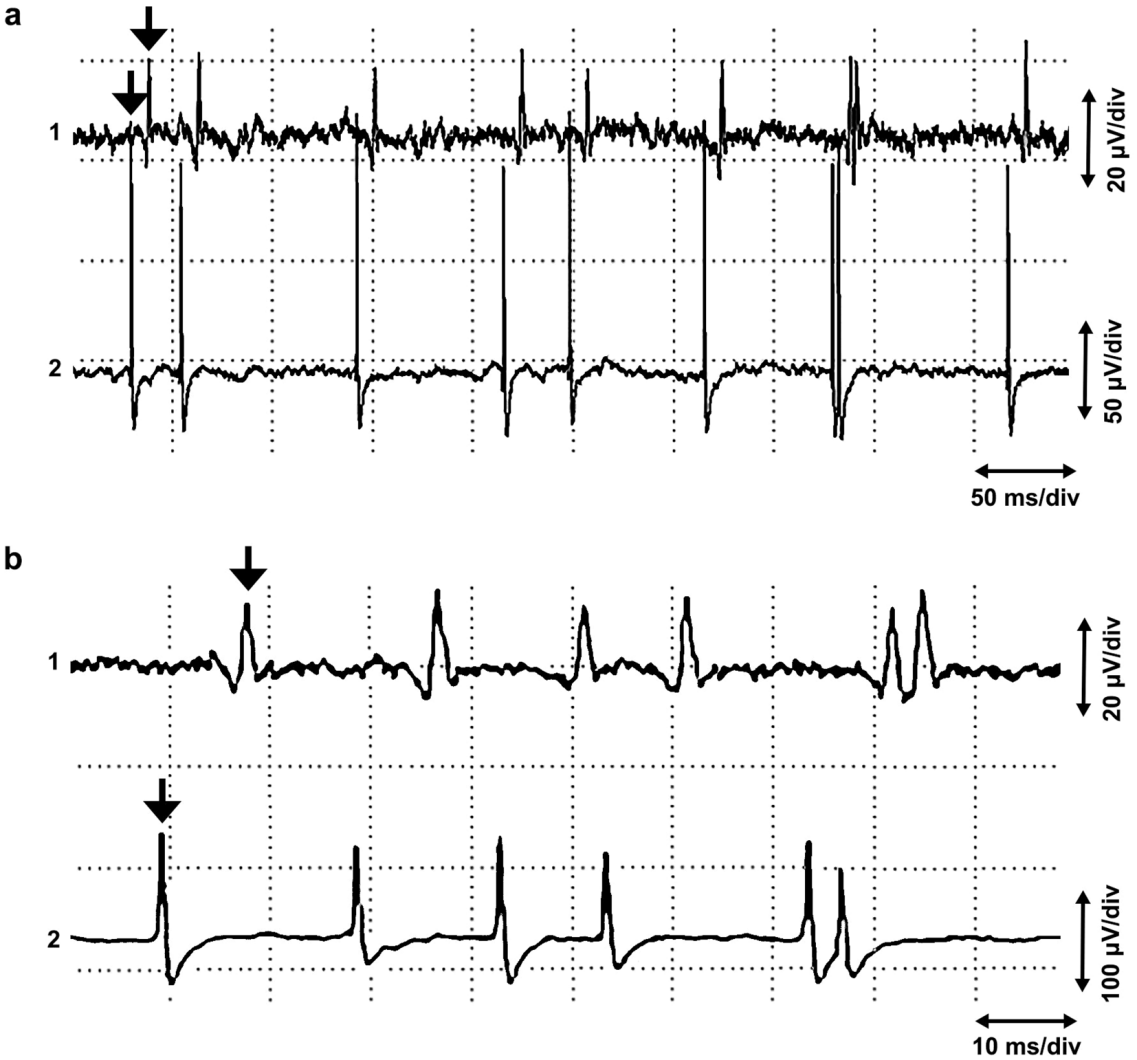
Exactly correlated end plate spikes in two active sites in different fasciculi in channels 1 and 2 (**a,b**) arrows, different time and amplitude calibration). The extensor carpi radialis muscle. The electrodes are inserted transversally with respect to the muscle fibers, interelectrode distance 4 mm. Note that there is a long interval, 8 ms, between exactly correlated potentials in channels 1 and 2.

**Figure 5:**
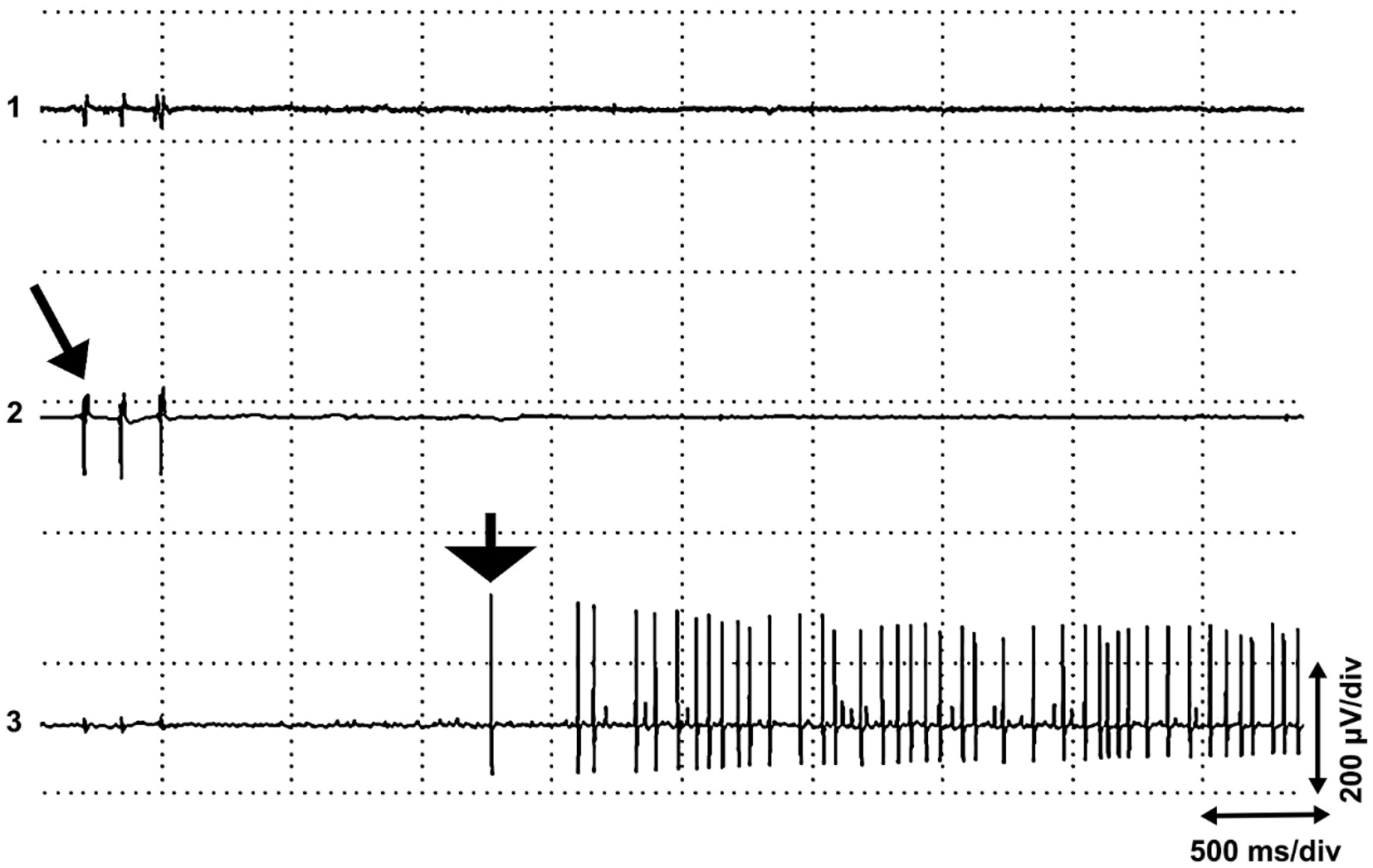
Activation of a sequence of end plate spikes. Passive stretching (slow extension of the forearm) of the muscle activates a sequence of local “end plate spikes” (channel 3, arrowhead). The last propagating motor unit potentials of the myotatic reflex are seen (tilted arrow), before the activation of end plate spikes in the biceps brachii muscle.

**Figure 6:**
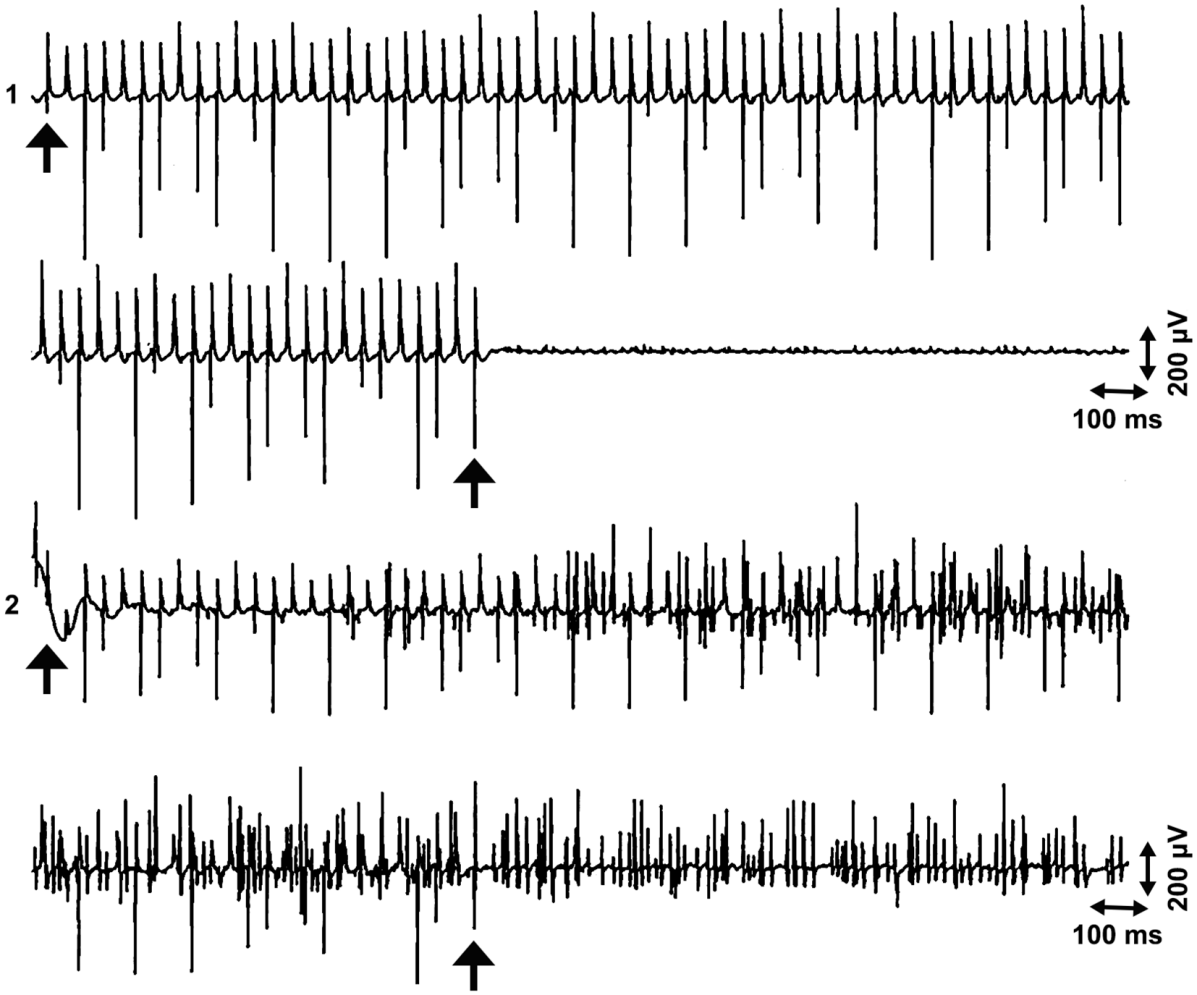
Activation of end plate spikes by high-frequency stimulation. Two-channel recording of the first interosseus dorsalis muscle. Channel 1: silent site; channel 2: active site with end plate spikes. High-frequency stimulation 30 Hz (start and stop, arrows; see stimulus artifact spikes), below the motor threshold, but above the sensory threshold, of the ulnar nerve at the wrist. No EMG activation at the silent site, but powerful activation of end plate spikes at the active site.

**Figure 7:**
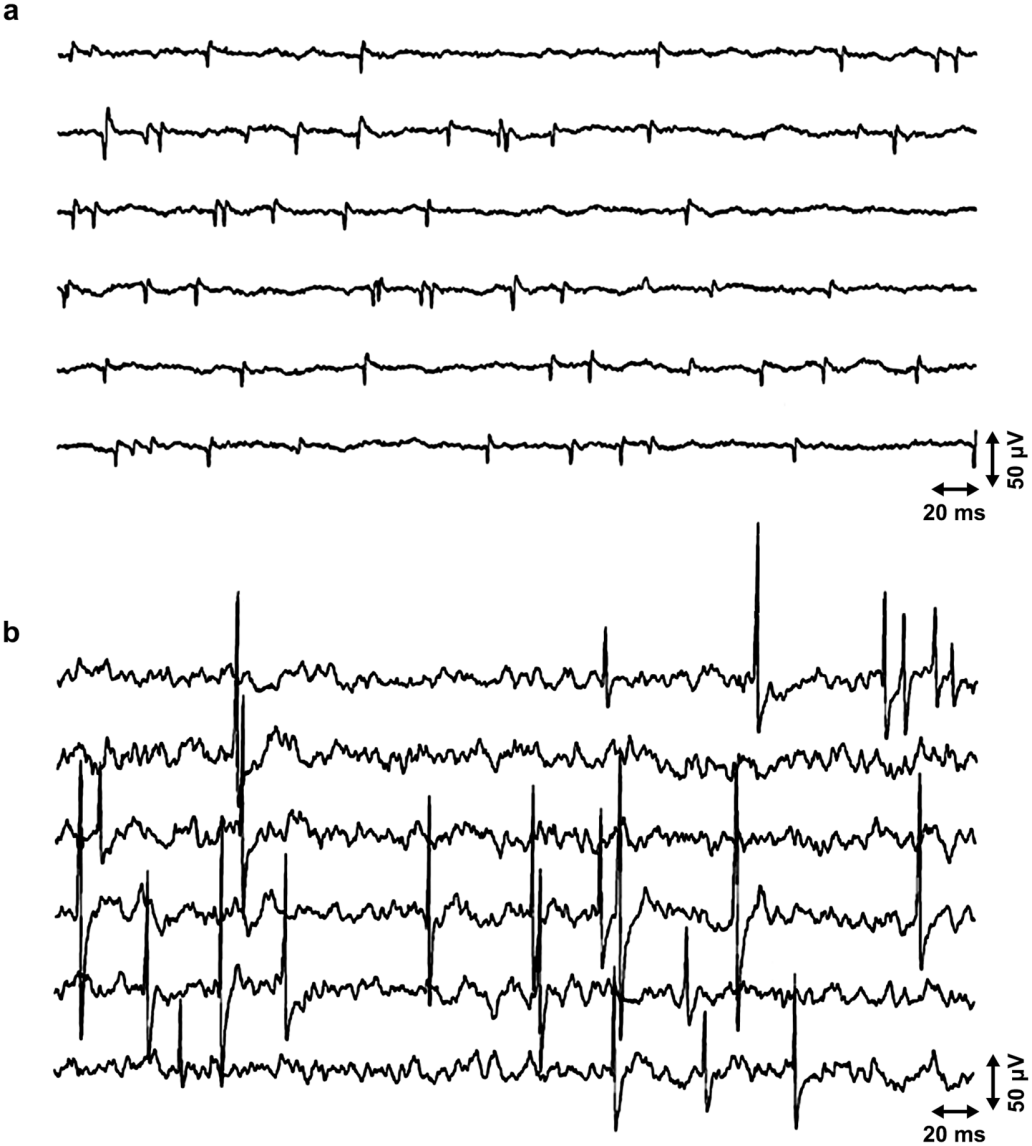
Sputtering positive potentials in the tibialis anterior muscle. (**a**) The concentric EMG needle penetrated through the active site, and the end plate spike potentials were recorded outside the active site as sputtering “cannula potentials”. (**b**) When the EMG needle was withdrawn to the active site, the waveform of “cannula potentials” changed to the usual appearance of end plate spikes. Note end plate noise in the background.

**Figure 8:**
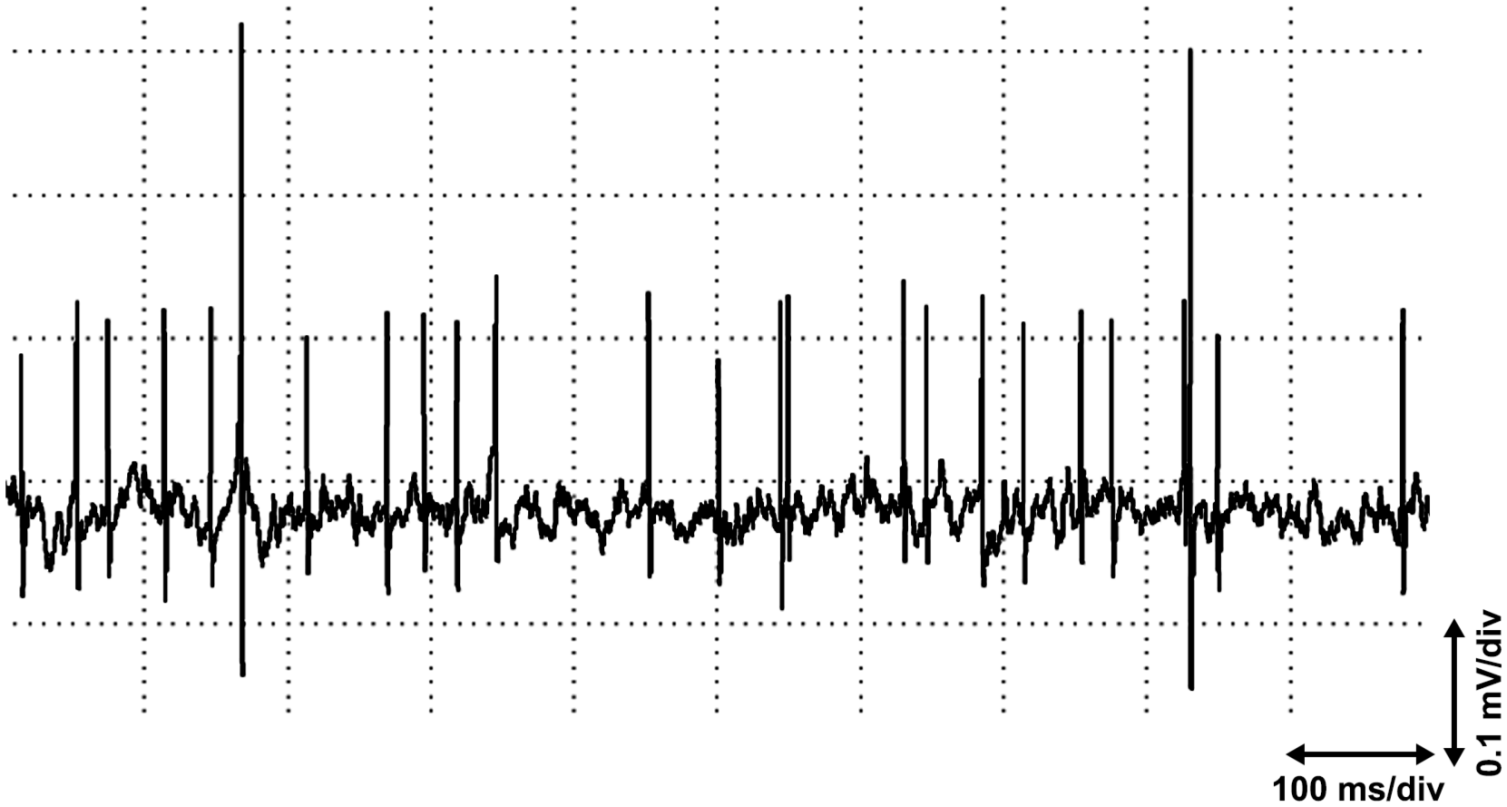
End plate spikes arising far from the end plate zone. End plate spikes recorded 4 cm proximal to the end plate zone (14 cm proximal to the distal tendon insertion of the muscle and 7 cm distal to the tendon of m. pectoralis major) in the short head of the biceps brachii muscle. End plate noise in the background.

The study was performed in a group of four voluntary healthy physicians of our laboratory, 2 female, age 32 and 59 years, and 2 male, age 28 and 70 years. There were no distinct differences in the EMG of “young” and “old” subjects. Figures 1b, 2, 4, 5 and 8 were recorded from “old” subjects, and Figs. 1a, 3, 6 and 7 from “young” subjects. We recorded needle EMG signals of the biceps brachii, extensor carpi radialis, interosseus dorsalis I, or tibialis anterior muscles in a resting condition without any fixation of the joints. The effect of passive stretch to the brachial biceps muscle was tested by extending the bowed forearm slowly. Two participants were tested. 1–5 small (Gauge 30, Ambu Neuroline) concentric EMG electrodes were inserted into the muscle and an intramuscular active site or sites with miniature end plate potentials and/or “end plate spikes” were searched with small insertions of the needles. Surface grounding was fixed near the recording site. With careful adjustment of the EMG needles in parallel with muscle fibers, propagated spontaneous action potentials of single muscle fibers can be recorded, such as needle insertion potentials^12^ and fibrillation potentials^13^. Also, both strictly local and propagated complex repetitive discharges, a rare abnormal waveform, as well as propagated motor unit potentials can be studied with this multi-channel EMG technique^14^. In the biceps brachii muscle, it was possible to study spontaneous EMG activity of the narrow motor innervation zone, as well as regions outside of it. The motor point was searched by stimulating the musculocutaneous nerve and looking for the negative onset of the recorded motor response with surface electrodes. The negative onset is the sign of the motor point^15^.

The propagation of spontaneous action potentials was studied by inserting the needle electrodes longitudinally relative to the muscle fibers in a fasciculus. The intramuscular interelectrode distance was approximately 2 mm. The position of the needles had to be tilted to different angles with the insertions because the sockets of the needle electrodes were 6 mm wide. Thereafter, the spreading of the potentials was studied by inserting a second needle to a different fasciculus with respect to the original needle with spontaneous activity, searching for exactly correlated activity with small insertions of the second needle. This technique revealed the possible neural pathway for spreading. In some cases, also a superficial skin electrode or a remote needle electrode was used to observe the propagation of motor unit potentials. The effect of high-frequency stimulation was studied at a silent and an active site of the interosseus dorsalis I muscle. The ulnar nerve was stimulated with 30 c/s electric pulses, duration 0.2 ms, for 5 s. The amplitude of the stimulation was adjusted below the motor threshold but above the sensory threshold, so that there were no M-responses but the stimulation could be sensed. The EMG potentials were recorded either with Keypoint (Alpine Biomed, Skovlunde, Denmark) or Synergy (Oxford Instruments Medical, Inc., UK) EMG equipment. High-pass and low-pass filters with cut-offs at 20 Hz and 10 kHz, respectively, were used. Electrically isolated preamplifiers were used with software-controlled interconnection of reference inputs. Sensitivity was 0.5 µV/div–20 mV/div (19 steps). Amplification was 20–200 µV/div according to the size of the potentials recorded.

The procedure was approved by the Ethics Committee of the Hospital District of Helsinki and Uusimaa. Each study participant gave her/his informed consent for the study, and all methods were performed in accordance with the relevant guidelines and regulations. By using the thinnest possible concentric EMG needle electrodes, the pain elicited by the insertions was well tolerated and none of the sessions were interrupted because of pain.

## Results

With small insertions of the EMG needle in the muscles studied, it was possible to find “active sites” with end plate spikes^4^. If several longitudinal electrodes were used, the most common finding was a local active site with a sequence of non-propagating end plate spikes and miniature end plate potentials or end plate noise in the background (Fig. 1a). Propagating end plate spikes were also found, but usually these propagated only a short distance, about 4 mm (Fig. 1b). Most end plate spikes, especially the non-propagating ones, had a single peak, but propagating one- or two-peaked end plate spikes were also observed (Fig. 2). A quiescent active site may at first show only miniature end plate potentials, while end plate spikes are activated after a long interval of up to 250 ms. At the end plate zone, it was possible to record very long sequences of miniature end plate potentials only, without end plate spikes (Fig. 3).

With two needle electrodes in different fasciculi, separate active sites with independently firing end plate spikes could be found with small insertions of the needles. But it was sometimes possible to observe exactly correlated end plate spikes in two different fasciculi (Fig. 4). In these cases, propagated potentials of a single muscle fiber are not possible. The time interval between the exactly correlated potentials in different localizations of the needle was relatively long, up to 8 ms.

Some activation procedures for end plate spikes were performed. Passive stretching of the relaxed muscle elicited myotatic reflexes but also activation of local end plate spikes (Fig. 5). Small bending of the needle could also activate end plate spikes. High-frequency stimulation had no effect at the silent site but activated end plate spikes at the active site. This activation continued, even after the stimulation was finished (Fig. 6).

If the concentric needle was inserted through an active point, end plate spikes with a positive waveform were recorded, but the waveform changed when the needle was withdrawn to the active point (Fig. 7). In the biceps brachii muscle, we could record end plate spikes at a site that was remote relative to the end plate zone (Fig. 8).

## Discussion

There are only two spontaneous waveforms in a healthy skeletal muscle, miniature end plate potentials and end plate spikes, if the insertion activity during needle insertions is not considered. Therefore, it is easy to recognize the description of these two waveforms in the literature, irrespective of the terms used. Miniature end plate potentials arise postsynaptically in the neuromuscular junction by a continuous small leakage of acetylcholine into the synaptic cleft^5^. The origin of end plate spikes is a more controversial issue.

Originally, end plate spikes, or “nerve potentials”, were conjectured to arise in intramuscular nerve filaments, caused by irritation of a nerve filament by a hit of the EMG needle in the muscle tissue, and recorded with the same needle^2^. The histological analysis with electrically induced iron dots at the active sites seemed to support the “nerve potential” hypothesis. The iron dots were often close to large-diameter nerve fibers. It was also claimed that iron dots were not found in muscle spindles^4^. However, the iron dots were put to the active sites using electric currents, which severed iron from the tip of the electrode. But muscle fibers contract and the intramuscular electrode moves if muscle fibers are stimulated with electric currents. This fact may have caused bias in the results of the histologic study using iron dots^10^. It may also be conjectured that the needle electrode stimulates nerve terminals directly, causing a release of acetylcholine vesicles and generating an end plate potential, which may reach the threshold for an action potential in the postsynaptic muscle fiber^6^ (Fig. 3). End plate spikes were subsequently proven undeniably to be postsynaptic^7^. The needle irritation of an intramuscular motor nerve fiber or nerve terminal was considered, most likely through physical contact, even if the recording is postsynaptic from the muscle fiber. This is the present, prevailing hypothesis^8^. However, an ectopic nerve action potential goes in both directions from its place of origin^16^. Thus, the action potential generated in the nerve filament should spread both orthodromically to the neuromuscular junction and antidromically to the whole motor unit^17^. As a consequence, a propagating motor unit potential or fasciculation potential should be observed, instead of an end plate spike^10^.

The theories described above, however, do not solve the appearance of end plate spikes, which are readily found far from the narrow end plate zone, as shown in the short head of the biceps brachii muscle^10^ (Fig. 8). The recording in Fig. 8 is from a region of the biceps brachii muscle with muscle spindles but with no extrafusal end plates of alpha motor neurons^18^. Muscle spindles are distributed in a wide area of the skeletal muscle^19^. Moreover, if end plate spikes are elicited locally at the end plate, or in a motor nerve twig, only one sequence of end plate spikes should be observed. It is not possible that several independent sequences of end plate spikes arise at only one site of irritation. We have shown that four different, independently firing sequences of end plate spikes can be recorded in an active site^9^. Moreover, we also observed that most end plate spikes are local (Fig. 1a), although also propagating ones can be found^10^ (Fig. 1b). This kind of dichotomy is not in concert with the present hypothesis, since extrafusal muscle fiber potentials will always propagate. The present hypothesis rules out the existence of strictly local, non-propagating end plate spikes (Fig. 1a). Irritation or injury of a motor nerve fiber causes only one or a few action potentials^20^, or rarely rhythmic activity, also known as “crazy units”^21^, but not sustained fast and randomly firing activity that is typical of end plate spikes^10^. With a little searching with small needle insertions, several active spots with independently firing end plate spikes can be found, for example, in the extensor carpi radialis muscle. End plate spikes are readily differentiated from fibrillation potentials of denervated or injured muscle fibers by their firing patterns and waveform^10,22,23^. The explanation for local and propagated end plate spikes may be found if we consider that there is dynamic and static gamma motor innervation of muscle spindles. Dynamic gamma efferents activate only non-propagating local junction potentials^11^. Static gamma efferents activate both local junctional potentials and propagating action potentials^11^.

The muscle spindle is the sensory receptor of muscle tissue, measuring both the length and change of length of a muscle^24^. The human muscle spindle is not very small, its average length is 5.8 mm, SD 1.86^25^. In order to keep its sensitivity when the length of the muscle changes, the muscle spindle needs a way to adjust its sensitivity. This is performed by the intrafusal muscle fibers, Ia afferents of the muscle spindle activate the stretch reflex but do not cause reflex activation of the fusimotor neurons^26^. Ia afferents are situated at the equatorial zone of the muscle spindle. In addition to Ia afferents, there are intrafusal II-afferents at the juxta-equatorial area^27^. II-afferents take part in the adjustment of fusimotor activity^24,28^. There are also intrafusal III- and IV-afferents, such as pain receptors and unmyelinated C-fibers^29–31^ activating the fusimotor units via the spinal reflex mechanism.

There are several types of intrafusal muscle fibers. The nuclear bag muscle fibers comprise two types: type 1 and type 2 fibers. The nuclear chain fibers comprise small chain fibers and a single long chain fiber. The type 1 bag fiber gets its innervation mainly from the dynamic gamma efferents^11^. The nuclear chain fibers are reminiscent of extrafusal muscle fibers. The nuclear bag 2 and nuclear chain muscle fibers get their innervation, with a few exceptions, via the static gamma motor neurons^11^. Both halves of the muscle spindle receive a separate motor innervation. Occasionally, a branch of a gamma fusimotor nerve fiber leaves the spindle capsule and supplies part of the fusimotor innervation of another spindle nearby^27^. Each human muscle spindle contains on average 8–20 intrafusal fibers^32^. Muscle spindles have cholinergic neuromuscular junctions of their own^24^. If every intrafusal muscle fiber gets motor innervation via a neuromuscular junction on both halves of the muscle spindle, there may be 16–40 neuromuscular junctions in one muscle spindle. An entering gamma motor nerve fiber branches and may end in a group of end plates on one or several intrafusal muscle fibers^27^. This fact may even increase the number of neuromuscular junctions inside the muscle spindle. In EMG of a muscle spindle, a great number of end plates with miniature end plate potentials and potentials of intrafusal muscle fibers may cause vivid activity after electric stimulation (see Fig. 6), evidently via different sensory afferents to the gamma efferent spinal reflex pathway in this case.

The average diameters of the nuclear bag 1 and bag 2 fibers are 18.4 and 19.9 µm, respectively, which are about 40% of that of extrafusal muscle fibers (41–59 µm)^33^. The nuclear chain fibers are thinner, on average 11.1 µm^25^, i.e., about 20% of the diameter of extrafusal muscle fibers, except the long chain fiber, which is bigger and resembles a thin extrafusal muscle fiber^24^. Thus, the intrafusal muscle fibers are large enough to be recorded with the conventional needle EMG. EMG electrodes are able to record even the non-propagating small postsynaptic miniature end plate potentials of the neuromuscular junction (Fig. 3)^5^. Consequently, we conclude that end plate spikes are potentials of fusimotor units^9,10^ and will use the term *fusimotor unit potential* or “end plate spike” in the following paragraphs. Muscle fibers lying within about one millimeter of the recording surface of the electrode make a significant contribution to the spike of a motor unit potential, and only 1 to 3 muscle fibers determine the peak amplitude of the motor unit potential^34^, in this case, the fusimotor unit potential. We think that fusimotor unit potentials together with miniature end plate potentials are recorded within the capsular region, where also the neuromuscular junctions are located. But we have also observed fusimotor unit potentials without miniature end plate potentials, and these may be recorded outside the end plate, for example, propagated potentials of the long chain fiber outside the capsular region or in the polar region of the muscle spindle, out of the neuromuscular junction zone.

The prevailing hypothesis states that “end plate spikes” originate only at the end plate or innervation zone of a muscle. It was even claimed that there is no spontaneous activity present outside the innervation zone^35^. It was considered that intrafusal muscle fibers are clearly another potential source for spontaneous activity outside the end plate zone^35^. Muscle spindles, however, are not preferentially in the region of the end plate zone for the extrafusal muscle fibers^35^. Thus, the biphasic spikes (end plate spikes) so characteristically recorded in close proximity to miniature end plate potentials must, therefore, arise in extrafusal muscle fibers^35^. But no other type of intrafusal spontaneous activity in EMG was ever described, except “end plate spikes”^9^. And it was not considered that intrafusal muscle fibers have also end plates with miniature end plate potentials. Intrafusal miniature end plate potentials were described in intrafusal recordings as follows: “spontaneous miniature potentials were observed, suggesting the micro-electrode might be close to activated motor terminals”^11^. In the biceps brachii muscle, “end plate spikes” may be found far from the narrow, about 1 cm wide end plate zone^36^ (Fig. 8).

There may be several active fusimotor units in one muscle spindle. A dynamic fusimotor unit gives rise to junctional potentials, as in dynamic bag 1 fibers (Fig. 1a). A static gamma motor unit may achieve a single- (Fig. 1b) or two-peaked waveform, where only one peak propagates (Fig. 2). The non-propagating peak may originate from a local junctional potential of a static bag 2 fiber, and the propagating one from a nuclear chain fiber^11^. But the junctional potential of the bag 2 fiber of the static gamma motor unit may also be solitary (Fig. 1a). If the EMG needle penetrates the active site to the extrafusal tissue, fusimotor unit potentials have a positive waveform, known as “sputtering cannula potentials”. But when the needle is withdrawn, the waveform changes^37^ (Fig. 7). Evidently, the electrode records intrafusal potentials. Each gamma and beta axon innervates several spindles but in a selective manner. If fusimotor unit potentials of a given unit are recorded from different muscle spindles, the interval of exactly correlated potentials may be long, indicating slow conduction in gamma motor nerve fibers (Fig. 4b). There is also physiological evidence of beta motor innervation in humans^38^. Widely propagating fusimotor unit potentials, evidently deriving from beta motor units, have also been observed^10,39^.

Characteristically, fusimotor unit potentials fire with fast irregular intervals, with numerous short intervals less than 30 ms^10^. The fast and irregular firing of fusimotor unit potentials could be caused by the lack of recurrent inhibition of gamma motor neurons^40^. This is different from the powerful recurrent inhibition of alpha motor neurons via Renshaw cells, and thus long interpotential intervals and a relatively regular firing pattern^41^. A needle penetrating the capsule of a muscle spindle presumably activates the sensory receptors of the thin afferent nerve fibers of the muscle spindle, comprising II-, III-, and IV-afferents, all of which activate the gamma motor units via the spinal reflex pathways. The latency of activation of fusimotor unit potentials in a quiescent muscle spindle after needle penetration, and observation of miniature end plate potentials, may be long, 250 ms^10^, which demonstrates that the reflex arc may be relatively slow. The afferent nerve fibers are either thinly myelinated or unmyelinated fibers from sensory receptors of the muscle spindle^30,31^, and the efferent motor fibers are small-diameter (4–8 µm) myelinated gamma motoneurons^42^. The great reflex responsiveness seems to be a feature of the gamma motor neurons.

Fusimotor unit potentials may have sustained firing for a long time, even minutes, although they normally slow down and eventually stop if the EMG needle is held still^10^. Thus, the reactions of fusimotor unit potentials may be studied. When a muscle is volitionally contracted, the alpha motor units are activated together with the gamma motor units^43^. However, in a relaxed muscle, fusimotor unit potentials may be activated in many ways, by penetrating the active spot (muscle spindle), tilting of the EMG needle^3^, passive stretching of the muscle (Fig. 5), or tetanic stimulation of the mixed nerve (Fig. 6), but not by H-reflex measurement, sympathetic activation (deep breathing), circulatory arrest, voluntary command, or Jendrassik maneuver^9,10^. The possibility to activate “end plate spikes” was disputed: “We have not noticed any alteration in the discharge frequency of the biphasic potentials, (end plate spikes) induced by moderate passive stretch or voluntary contraction of the muscle, such as to suggest these potentials were generated by the intrafusal muscle fibers”^35^. But with this statement, neither objective data nor figures were presented. Our findings with various activation procedures are opposite^10^. In order to study activation procedures of the fusimotor unit potentials, several recordings are needed because the recording electrode easily slips out of the active site if the muscle contracts or is moved. The expected reaction does not take place in every trial. The unquestionable activating effects of passive stretch and voluntary contraction were described with figures of the recordings^10^.

The potentials of neuromuscular junctions in the rat diaphragm and human muscles have been studied^7^. The observation that neuromuscular blocking agents blocked the “biphasic potentials” (end plate spikes) proved that these potentials must be postsynaptic^7^. The neuromuscular blocking agents block both the cholinergic alpha and gamma motor neuromuscular transmission, and if we assume that muscle spindles were studied, instead of neuromuscular junctions of alpha motor neurons, the observations with “biphasic negative–positive potentials” (end plate spikes) can be understood. The fine structure of the rat intrafusal muscle fibers is similar to the human ones^44^. There is a small number of muscle spindles in the rat diaphragm^45^. Muscle spindles are situated near the entry of the nerve trunk into the muscle and along the intramuscular nerves^24^. The rat hemidiaphragm preparation provides an excellent chance to test the origin of “biphasic negative–positive potentials”, because the end plate zone could be located by transillumination from below^7^. But this zone is in close proximity to the main trunks of the phrenic nerve^46^. Evidently, there are also muscle spindles intermingled with the end plates of the alpha motor neurons, and intrafusal neuromuscular junctions cannot be distinguished from the extrafusal ones in the recording.

Because muscle spindles contain a large number of intrafusal neuromuscular junctions, we may assume that intrafusal miniature end plate potentials and fusimotor unit potentials (“end plate spikes”) were studied instead of miniature end plate potentials of alpha motor neurons^7^. Our experience when performing routine EMG is that miniature end plate potentials with “end plate spikes” are far more readily found than miniature end plate potentials without “end plate spikes”, produced by a neuromuscular junction of an alpha motor neuron at the end plate zone only. This is conceivable because neuromuscular junctions are heavily concentrated in muscle spindles, which are large (6 mm)^25^ and in all parts of the muscle, compared to scattered minute extrafusal neuromuscular junctions (50 µm)^47^ at the end plate area. Thus, intrafusal miniature end plate potentials with fusimotor unit potentials, “end plate spikes”, are readily found with needle insertions in clinical EMG studies^4^. This holds true also for the human gastrocnemius, where the neuromuscular transmission block was studied by neuromuscular blocking agents^7^. Locations in the end plate zone where miniature end plate potentials and “biphasic action potentials” (end plate spikes) had the maximum voltage were sought^7^. But this means that the end plate zone was falsely located according to the intrafusal miniature end plate potentials and fusimotor unit potentials of *any* muscle spindle. One individual muscle fiber is only a small percent of the total gastrocnemius muscle length. Consequently, the end plate zone runs centrally down the length of each compartment of the muscle^47^.

Extrafusal alpha neuromuscular junctions express only miniature end plate potentials or end plate noise, but no end plate spikes at all^10^. We have recorded long sequences of miniature end plate potentials without “end plate spikes” at the end plate zone (Fig. 3). The usual way to assess fusimotor activity indirectly by using microneurography of the afferent nerve fibers of the muscle spindles^43^ might also be supplemented by the direct EMG recording of fusimotor unit potentials, especially in resting conditions. Voluntary motor activity masks fusimotor unit potentials, but they usually continue firing after sudden cessation of voluntary effort^10^. EMG might also complement the difficult method to record efferent gamma motor nerve fibers of peripheral nerves with microneurography^48^. An easy way to map the existence of muscle spindles in different muscles or in different parts of a muscle may be to study the appearance of active sites with spontaneous fusimotor unit potentials with EMG. It may also be possible to study the physiology and pathophysiology of muscle spindles in muscle pain syndromes^39,49^. In fibromyalgia, the EMG marker of a painful trigger point^50^ is similar to the pattern of activation of fusimotor unit potentials in an active site (Fig. 5). This indicates the role of muscle spindles in the formation of trigger points. The muscle spindles have a rich palette of different EMG activities, not observed in the silent, extrafusal site of the muscle. These activities should be studied experimentally, and in health and disease.

## References

[1] Snodgrass JM, Sperry RW (1941). Mammalian muscle action potentials of less than a millisecond (Abstract). Am. J. Physiol..

[2] Jasper H, Ballem G (1949). Unipolar electromyograms of normal and denervated human muscle. J. Neurophysiol..

[3] Kugelberg E, Petersen I (1949). “Insertion activity” in electromyography. J. Neurol. Neurosurg. Psychiatry.

[4] Jones RV, Lambert EH, Sayre GP (1955). Source of a type of “insertion activity” in electromyography with evaluation of a histologic method of localization. Arch. Phys. Med. Rehabil..

[5] Wiederholt WC (1970). “End-plate noise” in electromyography. Neurology.

[6] Buchthal F, Rosenfalck P (1966). Spontaneous electrical activity of human muscle. Electroencephalogr. Clin. Neurophysiol..

[7] Brown WF, Varkey GP (1981). The origin of spontaneous electrical activity at the end-plate zone. Ann. Neurol..

[8] Dumitru D (2000). Physiological basis of potentials recorded in electromyography. Muscle Nerve.

[9] Partanen JV, Nousiainen U (1983). End-plate spikes in electromyography are fusimotor unit potentials. Neurology.

[10] Partanen J (1999). End plate spikes in the human electromyogram: Revision of the fusimotor theory. J. Physiol. Paris.

[11] Barker D, Bessou P, Jankowska E, Pagès B, Stacey MJ (1978). Identification of intrafusal muscle fibres activated by single fusimotor axons and injected with fluorescent dye in cat tenuissimus spindles. J. Physiol..

[12] Daube JR (1991). AAEM minimonograph #11: Needle examination in clinical electromyography. Muscle Nerve.

[13] Partanen J, Lang H, Häkkinen V, Larsen A, Partanen J (1991). Spontaneous activity. Our Electric Nerves, Textbook of Electroneuromyography.

[14] Partanen JV (2016). Ephaptic transmission from type II afferents to static γ and β efferents causes complex repetitive discharge: An hypothesis. Muscle Nerve.

[15] Saitou K, Matsuda T, Michikami D, Kojima R, Okada M (2000). Innervation zones of the upper and lower limb muscles estimated by using multichannel surface EMG. J. Hum. Ergol..

[16] Rasminsky M (1980). Ephaptic transmission between single nerve fibres in the spinal nerve roots of dystrophic mice. J. Physiol..

[17] Stålberg E, Trontelj JV, Culp WJ, Ochoa J (1982). Abnormal discharges generated within the motor unit as observed with single-fibre electromyography. Abnormal Nerves and Muscles as Impulse Generators.

[18] Aquilonius SM (1984). Topographical localization of motor endplates in cryosections of whole human muscles. Muscle Nerve.

[19] Radziemski A, Kedzia A, Jakubowicz M (1991). Number and localization of the muscle spindles in the human fetal sternocleidomastoid muscle. Folia Morphol..

[20] Wall PD, Waxman S, Basbaum AI (1974). Ongoing activity in peripheral nerve: injury discharge. Exp. Neurol..

[21] Macefield VG (1998). Spontaneous and evoked ectopic discharges recorded from single human axons. Muscle Nerve.

[22] Partanen JV, Danner R (1982). Fibrillation potentials after muscle injury in humans. Muscle Nerve.

[23] Partanen J, Turker H (2011). Different types of fibrillation potentials in human needle EMG. Electrodiagnosis.

[24] Banks RW, Barker D, Engel AG, Franzini-Armstrong C (2004). The muscle spindle. Myology.

[25] Sahgal V, Subramani V, Sahgal S, Kochar H, Boyd IA, Gladden MH (1985). Morphology and morphometry of human muscle spindles. The Muscle Spindle.

[26] Appelberg B, Hulliger M, Johansson H, Sojka P (1983). Actions on gamma-motoneurones elicited by electrical stimulation of group I muscle afferent fibres in the hind limb of the cat. J. Physiol..

[27] Swash M, Fox KP (1972). Muscle spindle innervation in man. J. Anat..

[28] Appelberg B, Hulliger M, Johansson H, Sojka P (1983). Actions on gamma-motoneurones elicited by electrical stimulation of group II muscle afferent fibres in the hind limb of the cat. J. Physiol..

[29] Stacey MJ (1969). Free nerve endings in skeletal muscle of the cat. J. Anat..

[30] Abrahams VC (1986). Group III and IV receptors of skeletal muscle. Can. J. Physiol. Pharmacol..

[31] Lund JP (2010). Assessment of the potential role of muscle spindle mechanoreceptor afferents in chronic muscle pain in the rat masseter muscle. PLoS ONE.

[32] Kröger S, Watkins B (2021). Muscle spindle function in healthy and diseased muscle. Skelet. Muscle.

[33] Dubowitz V, Brooke MH (1973). Muscle Biopsy: A Modern Approach.

[34] Stålberg E, Trontelj JV, Sanders DB (2010). Single Fibre Electromyography.

[35] Brown WF, Brown WF (1984). Normal and abnormal spontaneous activity in muscle. The Physiological and Technical Basis of Electromyography.

[36] Amirali A, Mu L, Gracies JM, Simson D (2007). Anatomical localization of motor endplate bands in the human biceps brachii. J. Clin. Neuromusc. Dis..

[37] Pickett JB, Schmidley JW (1980). Sputtering positive potentials in the EMG: An artifact resembling positive waves. Neurology.

[38] Kakuda N, Miwa T, Nagaoka M (1998). Coupling between single muscle spindle afferent and EMG in human wrist extensor muscles: Physiological evidence of skeletofusimotor (beta) innervation. Electroencephalogr. Clin. Neurophysiol..

[39] Partanen JV, Watkins M, Hsüeh L (2017). Muscle spindles and beta motor units in trigger point and taut band formation. Trigger Points: Etiology, Pathophysiology and Clinical Management.

[40] Hunt CC, Paintal AS (1958). Spinal reflex regulation of fusimotor neurones. J. Physiol..

[41] Eccles JC, Eccles RM, Iggo A, Ito M (1961). Distribution of recurrent inhibition among motoneurones. J. Physiol..

[42] Macefield VG, Knellwolf TP (2018). Functional properties of human muscle spindles. J. Neurophysiol..

[43] Vallbo ÅB (1971). Muscle spindle response at the onset of isometric voluntary contraction in man: Time difference between fusimotor and skeletomotor effects. J. Physiol..

[44] Ovalle WK (1971). Fine structure of the rat intrafusal muscle fibers: The polar region. J. Cell Biol..

[45] Barstad JAB, Kristoffersen A, Lilleheil G, Staaland H (1965). Muscle spindles in the rat diaphragm. Experientia.

[46] Corda M, von Euler C, Lennerstrand G (1965). Proprioceptive innervation of the diaphragm. J. Physiol..

[47] Mense S, Simons DG, Mense S, Simons DG (2001). Location of motor endplates: Neuromuscular junction. Muscle Pain.

[48] Ribot E, Roll JP, Vedel JP (1986). Efferent discharges recorded from single skeletomotor and fusimotor fibres in man. J. Physiol..

[49] Partanen JV, Ojala TA, Arokoski JPA (2010). Myofascial syndrome and pain: A neurophysiological approach. Pathophysiology.

[50] Ge HY, Wang Y, Danneskiold-Samsøe B, Graven-Nielsen T, Arendt-Nielsen L (2010). The predetermined sites of examination for tender points in fibromyalgia syndrome are frequently associated with myofascial trigger points. J. Pain.

